# Research and Development of a High-Temperature-Resistant, Gel-Breaking Chemical Gel Plugging Agent and Evaluation of Its Physicochemical Properties

**DOI:** 10.3390/gels11050350

**Published:** 2025-05-08

**Authors:** Junwei Fang, Jinsheng Sun, Xingen Feng, Lijuan Pan, Yingrui Bai, Jingbin Yang

**Affiliations:** 1 School of Petroleum Engineering, China University of Petroleum (East China), Qingdao 266580, China; 2 Northwest Petroleum Branch, Sinopec, Urumqi 830011, China; 3 Key Laboratory of Enhanced Oil Recovery for Fractured-Vuggy Reservoirs, Sinopec, Urumqi 830011, China

**Keywords:** chemical gel lost circulation agent, high-temperature-resistance, gel-breaking, crosslinking agent, physical-chemical properties

## Abstract

Gas channeling phenomena in carbonate fracture-vuggy reservoirs frequently occur, primarily in the form of negative pressure gas channeling and displacement gas channeling, with the possibility of mutual conversion between the two. This is accompanied by the risk of hydrogen sulfide (H_2_S) release from the reservoir, which poses significant challenges to controlling safety. Currently, liquid bridging and gel plugging technologies are effective methods for mitigating complex issues such as downhole overflow, fluid loss, and heavy oil backflow. This paper focuses on the development and optimization of key treatment agents, including high-temperature-resistant polymers and crosslinking agents, to formulate a high-temperature chemical gel plugging agent. A gel-breaking, high-strength colloidal chemical gel plugging agent system capable of withstanding temperatures up to 150 °C was developed, and it has an apparent viscosity of about 7500 mPa·s, an energy storage modulus and a loss modulus of 51 Pa and 6 Pa, respectively, after gel formation at elevated temperatures, and an apparent viscosity retention rate of the gel of greater than 82% after aging for 9 d at a temperature of 150 °C. This system forms a stable gas isolation barrier in the wellbore, with performance remaining stable after 7 to 12 days of aging, and the degradation rate reaches 99.8% after 24 h at 150 °C. This technology is of significant importance in solving complex issues such as overflow, fluid loss, and heavy oil backflow in gas injection and recovery wells in high-temperature, high-pressure reservoir conditions.

## 1. Introduction

Chemical gel plugging agents, as a key displacement control technology, selectively plug high-permeability fractures, forcing subsequent displacement fluids to move toward unimpacted areas. This has become an important method to improve the development of heterogeneous reservoirs [1,2]. Negative pressure gas channeling is when the flow pressure at the bottom of the well is less than the formation pressure, and the gas in the formation will enter the wellbore under the effect of pressure difference. Displacement gas channeling mainly occurs in natural fractured formations. This type of formation is generally narrow, or even zero, and belongs to the category of spraying and leaking together with the coexistence of a narrow safe density window; therefore, there needs to be a chemical plugging agent for timely plugging. Traditional chemical gel plugging agents mainly include polymer gels, particle-based plugging agents, and foam systems [3,4,5]. Among these, polymer gels (such as polyacrylamide/phenolic resin systems) are widely used due to their high viscoelasticity and controllable gelling time. However, they are prone to degradation under high temperature (>90 °C) or high salinity (>20,000 ppm) conditions [6]. Particle-based plugging agents (such as nanosilica or pre-crosslinking gel particles) have good temperature and salt resistance, but their injectability and compatibility with reservoir pore structures remain challenging [7]. Foam systems, with their low flowability due to gas–liquid phase interaction, can achieve dynamic plugging, but their stability is significantly affected by surfactant properties and reservoir wettability [8]. Therefore, there is an urgent need to develop multifunctional plugging agent systems that can adapt to complex reservoir conditions.

As medium-to-shallow oil and gas resources continue to deplete, deep and ultra-deep oil and gas resources are receiving more attention. During deep and ultra-deep drilling and production, high temperature is one of the major challenges faced by drilling fluids [9]. For oil and gas drilling using polymer gel materials, polymer degradation at high temperatures leads to a decrease in gel strength, thus affecting their application effectiveness [10]. To address the high-temperature challenges of polymer gels, numerous studies have been conducted. Current research primarily focuses on optimizing the polymer molecular structure, improving crosslinking agent types, and adding high-temperature additives to gel compositions [11]. AMPS, due to its large side chains and sulfonic groups, is a common high-temperature monomer used for preparing single-component gels or copolymers with polymers. The copolymerization of AM with AMPS results in a copolymer with good thermal stability due to the steric hindrance provided by the methylpropane and sulfonic groups on the polymer molecular chains. Chen et al. [12] crosslinked the AM/AMPS copolymer with phenolic crosslinking agents and used ethylenediamine as a crosslinking retardant to prepare a gel with a dense three-dimensional network structure. This gel maintained good strength and stability after aging for 100 days at 130 °C, and was successfully applied for reservoir water shutoff operations in Sinopec Northwest Oilfield. Conventional plugging materials cannot fully meet the technical requirements of drilling loss plugging operations. Wang et al. [13] synthesized a high-temperature, salt-resistant polymer, HDZ-A, and by adding a certain concentration of crosslinking agent and retarder, they prepared a high-temperature delayed crosslinking polymer gel plugging agent. This gel maintained stability after high-temperature crosslinking and could withstand up to 160 °C. The gelling time reached 4.5 h under 160 °C, with a plugging efficiency exceeding 97%. Long et al. [14] prepared a high-temperature-resistant pre-crosslinking gel system by polymerizing acrylamide (AM) with cyclic N-vinylpyrrolidone (NVP) and divinylbenzene (DVB). After aging for 90 days at 130 °C, the gel maintained stable strength. Zhu et al. [15] reacted a ternary polymer of AM/AA/AMPS with a novel crosslinking agent, OC-3, which has hydroxyl and phenolic rings, to prepare a thermally stable, gel strength-adjustable underground crosslinking gel system. The amide group (−CONH_2_) on the ternary copolymer molecular chains crosslinks with the hydroxyl group (−CH_2_OH) on the phenolic ring of OC-3 to form a dense three-dimensional network structure. This system remained free of dehydration even after 5 months at 150 °C.

Crosslinking agents are essential components in the preparation of crosslinking polymer gels, and common crosslinking agents for polymer gels include organic phenolic resins and metal ions [16,17]. Due to the hydrolysis of metal ion-based crosslinking gels at high temperatures, leading to dehydration of the gel, the covalent bonds formed between the amide groups of the polymer and organic crosslinking agents are more stable at high temperatures, providing better thermal stability for the gels [18]. For single-monomer crosslinking polymer gels, N,N′-methylenebisacrylamide and N-hydroxyethylacrylamide are commonly used crosslinking agents. Niu et al. [19] successfully synthesized a mechanically strong, thermally stable supramolecular polymer gel using N,N′-methylenebisacrylamide as a crosslinking agent. This gel exhibited excellent salt resistance under harsh reservoir conditions. Compared to toxic phenolic crosslinking agents, organic polymer crosslinking agents, such as polyethyleneimine (PEI), are non-toxic and environmentally friendly. The crosslinking reaction between PEI and polyacrylamide forms stable covalent bonds by the nucleophilic imine nitrogen on PEI replacing the amide group on polyacrylamide. Therefore, PEI-crosslinking gels offer better high-temperature stability than metal ion crosslinking gels [20]. To solve the problem of dehydration of metal-ion crosslinking gels at high temperatures, organic–inorganic composite crosslinking agents are commonly used to enhance the tightness of the three-dimensional network structure, thus improving gel thermal stability. Zhang et al. [21] used Cr^3+^ and phenolic resin as crosslinking agents to prepare a composite crosslinking polyacrylamide gel system. This system contained both weak bonds from metal crosslinking agents and strong bonds from organic crosslinking agents. The existence of double bonds in the network effectively suppressed the dehydration of the metal crosslinking structure at high temperatures, significantly improving gel thermal stability. This gel remained stable even after 120 days of aging at 140 °C. To address the challenges of low polymer gel strength and poor stability in high-temperature, low-permeability reservoirs, Yang et al. [22] developed a new organic/metal ion composite crosslinking polymer gel (AR-Gel) for CO_2_-EOR applications. In this gel, Al(III) ions crosslinking with carboxyl groups and organic/metal ion double crosslinking builds a more complex and stable gel structure compared to conventional chemical crosslinking, allowing the gel to withstand harsh conditions such as high temperature.

Formation waters in oil and gas fields contain a certain concentration of salt ions, ranging from thousands to hundreds of thousands [23]. For polymer gels, the effect of salt on gel stability is mainly due to high concentrations of metal ions, especially divalent and trivalent ions, which compress the hydrophilic groups on the polymer molecular chains, causing the gel structure to curl and dehydrate, thereby significantly reducing gel strength [24]. Conventional high-molecular-weight polyacrylamide gels show poor salt resistance, as high ion concentrations severely compress the carboxyl and amide groups on molecular chains, forcing the chains to curl and shrink. Furthermore, high-valent cations (Ca^2+^, Fe^3+^, etc.) tend to complex with carboxyl groups, reducing crosslinking sites and decreasing gel stability [25,26]. Compared to high-molecular-weight polyacrylamide (typically > 10,000 kDa), low-molecular-weight polyacrylamide has better deformability and solubility, maintaining a good molecular shape under high-salinity conditions without excessive curling. Moreover, low-molecular-weight polyacrylamide undergoes more intermolecular crosslinking with crosslinking agents, which helps improve crosslinking density [27]. Fang et al. [28] used low-molecular-weight hydrolyzed polyacrylamide (molecular weight 3800 kDa) as the polymer and hexamethylenetetramine (HMTA)/hydroquinone (HQ) as the crosslinking agents to prepare a salt-resistant gel system that maintained over 90% plugging efficiency for high-permeability cores at a salinity of 19.8 × 10^4^ mg/L. The molecular structure of the polymer has an important effect on gel performance. Conventional linear polymers tend to curl under high-salinity conditions, while star-shaped polymers, composed of a central star-shaped core and multiple hyperbranched polymer chains, have enhanced molecular entanglement and better salt resistance than linear polymers [29]. Nie et al. [30] grafted β-cyclodextrin functional monomers onto AM copolymers, then hydrolyzed the copolymer to prepare a salt-resistant star-shaped polymer (Star-PAM). In subsequent research, the star-shaped polymer was crosslinking with a high-temperature-resistant crosslinking agent to develop a thermally stable, salt-resistant gel system that maintained a viscosity of 43,500 mPa·s after 180 days at 126 °C and a salinity of 117,000 mg/L. This gel, combined with a zwitterionic surfactant composite absorption technology, was tested in high-temperature, high-salinity reservoirs, achieving a cumulative oil production increase of 3660 tons.

To address the poor high-temperature performance, weak thixotropy, and difficulty in gel breaking of existing gel materials for gas loss plugging, this study aims to enhance gel crosslinking density and strengthen the skeleton structure by developing a new high-temperature crosslinking agent to replace conventional products. The combination of the high-temperature-resistant crosslinking agent with high-temperature-resistant polymers significantly improves the thermal stability of the polymer gels. Additionally, resin curing agents are selected to improve gel solution thixotropy and adjust solution flow characteristics, resulting in gels with viscoelastic and thixotropic responses. These gels achieve efficient drainage, self-filling, and high-strength gelling in three-dimensional spaces. By combining the new high-temperature crosslinking agents, resin curing agents, and high-temperature-resistant polymers, the gels demonstrate remarkable thixotropy before gelling, excellent stability after high-temperature gelling, and superior gel-breaking performance after plugging.

## 2. Construction of Chemical Gel Plugging Agent System

### 2.1. Selection of Polymer Type and Concentration

#### 2.1.1. Molecular Weight and Hydrolysis Degree of Different Types of Polymers

The hydrolysis degree of PAM is directly related to the proportion of carboxyl groups in the molecular structure [31]. The higher the hydrolysis degree of the polymer, the more carboxyl groups (−COOH) it contains. For inorganic crosslinking agents, they primarily react with the carboxyl groups on polyacrylamide. The higher the hydrolysis degree of the polymer, that is, the more carboxyl groups (−COOH) it has, the faster the gelling time, the greater the gelling strength, and the gel’s stability will have an optimal value. For organic crosslinking agents (phenol-formaldehyde systems and other polymer-based crosslinking agents), they primarily react with the amide groups (−COHN_2_) on polyacrylamide. The hydrolysis degree of the polymer is typically between 10% and 30%, so the effect of changes in the amide group is relatively minor on gelling performance. However, if the hydrolysis degree of the polymer is too low, it will inhibit the polymer’s hydration process, thereby prolonging the gelling time. It is important to note that if the hydrolysis degree is too low, the dissolution time of the polymer will also increase, which will lead to longer construction time and higher costs. In this study, the polymers were prepared by a terpolymerization process of acrylamide (AM), 2-acrylamido-2-methylpropanesulfonic acid (AMPS), and N-vinylpyrrolidone (NVP). Based on the above analysis, the different types of polymers selected in this study, ZP-1 and ZP-2, are summarized in Table 1.

#### 2.1.2. Salt Resistance of Different Types of Polymer Solutions

In the complex fluid environment of deep reservoirs, formation water mineralization has a significant influence on the formation process of subsurface crosslinking gel networks. Multivalent cations (Ca^2+^, Na^+^) weaken the bilayer repulsive force on the surface of polymer chain segments through electrostatic shielding, which changes the polymer molecular chain from a stretched random nematic conformation to a compacted globular conformation. This not only leads to a decrease in the viscosity of the solution, but also leads to a decrease in the effective collision frequency factor between the polymer molecules and the crosslinking agent, and a decrease in the effective active sites for crosslinking, which slows down the crosslinking process and affects the densification of the gel network structure [32].

**Figure 1:**
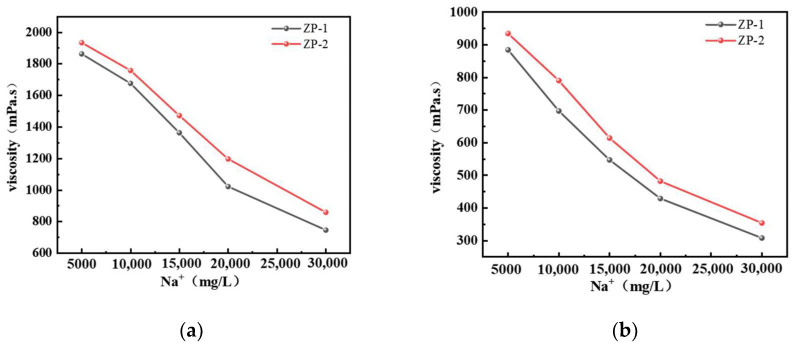
Effect of Na^+^ concentration on the viscosity of different types of polymer solutions: (**a**) 25 °C; (**b**) 150 °C.

The salt resistance of different types of polymers at a concentration of 1% was compared using a Brookfield viscometer under ambient temperature and 150 °C conditions. The selected Na^+^ concentrations were 5000, 10,000, 15,000, 20,000, and 30,000 mg/L. Figure 1 shows the effect of Na^+^ concentration on the viscosity of different types of polymer solutions. Polymer solutions with different mineralization levels were prepared using deionized water. After 24 h of aging at ambient temperature, the ZP-1 polymer solution exhibited relatively high viscosity, with values of 1862, 1675, 1362, 1021, and 745 mPa·s as Na^+^ concentration increased. After 24 h of aging at 150 °C, the viscosity of the polymer solution significantly decreased, with values of 884, 697, 547, 429, and 308 mPa·s as Na^+^ concentration increased. After 24 h of aging at ambient temperature, the viscosity of ZP-2 polymer solution increased with Na^+^ concentration, with values of 1933, 1757, 1471, 1196, and 858 mPa·s. After 24 h of aging at 150 °C, the viscosity significantly decreased, with values of 934, 790, 614, 482, and 354 mPa·s as Na^+^ concentration increased. After 24 h of aging at ambient temperature, the viscosity retention rates of ZP-1 and ZP-2 polymer solutions at Na^+^ concentration of 30,000 mg/L were 40% and 44%, respectively. Even at 150 °C and Na^+^ concentration of 30,000 mg/L, the viscosity retention rates of ZP-1 and ZP-2 solutions after 24 h of aging were 34% and 38%, respectively. From this, it can be observed that the temperature-resistant polymer exhibits better salt resistance compared to conventional polymers. This is primarily because the temperature-resistant polymer introduces the AMPS monomer, which imparts large side-chain groups to the polymer molecules, forming substantial steric hindrance. Additionally, the polymer contains -SO_3_- groups, contributing to a certain degree of salt resistance.

#### 2.1.3. TemperatureResistance of Different Polymer Solutions

**Figure 2:**
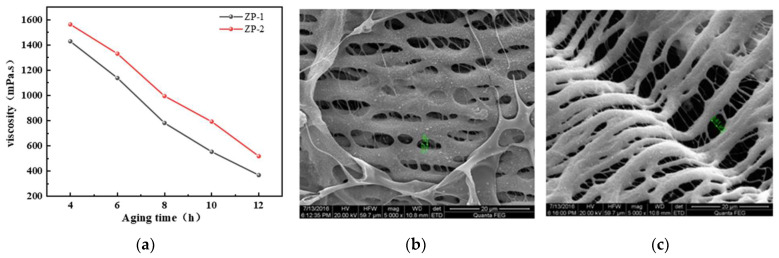
(**a**) Effect of temperature on the viscosity of different polymer solutions; (**b**) scanning electron micrograph of 1% ZP-2 polymer solution before aging; (**c**) scanning electron micrograph of 1% ZP-2 polymer solution after aging at 180 °C for 8 h. (Green color indicates the size of the hole at the same scale.)

Formation temperature is one of the key factors influencing the gelling performance of polymer gel systems [33]. The temperature resistance of polymer solutions ZP-1 and ZP-2 was evaluated using a Brookfield viscometer. As shown in Figure 2a, the viscosity of ZP-1 solution decreases significantly. After aging at 180 °C for 12 h, the viscosity retention is only 23% of the original polymer viscosity. In contrast, the viscosity of ZP-2 solution exhibits a smaller change. After aging at 180 °C for 12 h, the viscosity of ZP-2 solution drops to 31% of the original viscosity. After 8 h of high-temperature aging, the viscosity of the copolymer ZP-2 solution is higher than that of the copolymer ZP-1 solution. This indicates that the additional NVP monomer added to the copolymer enhances the thermal stability of the polymer, primarily because of the presence of thermally stable rigid groups in the NVP structure, which form larger spatial steric hindrance and inhibit the hydrolysis of the amide groups. This structure can significantly improve the temperature resistance of the polymer solution. As shown in Figure 2b,c, during the polymer sample preparation process, liquid nitrogen freeze-drying ensures that the polymer’s morphology is preserved as much as possible in the solution. Prior to aging, the polymer chains are densely distributed in the solution, mainly in a linear form, with a small amount of polymer distributed in fibrous form between the polymer chains. After aging the polymer solution at 180 °C for 4 h, some polymer chains break due to the combined effects of high temperature and dissolved oxygen. The distance between the polymer chains slightly increases, and the distribution of the polymer becomes slightly sparser. However, the distribution remains fairly intact, and as a result, the polymer ZP-2 maintains a high apparent viscosity even after 8 h of aging at 180 °C.

#### 2.1.4. The Effect of Polymer Blends on Viscosity

**Figure 3:**
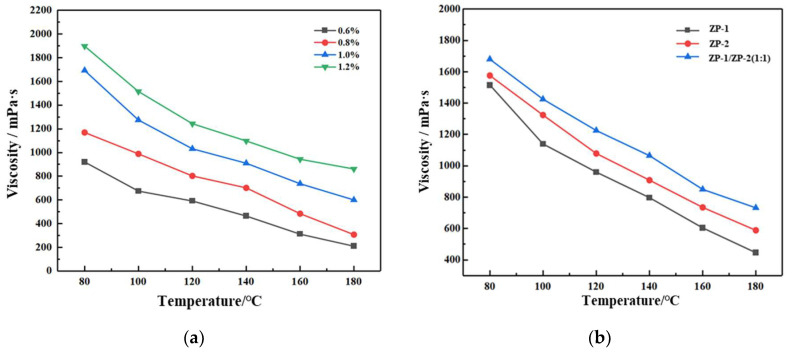
Viscosity–temperature curves: (**a**) viscosity–temperature curve for ZP-1: ZP-2 = 1:1 blend; (**b**) comparison of viscosity–temperature curves for single polymers and blended polymers at a 1% mass concentration.

Due to the high temperature (up to 180 °C) and high salinity of the formation water in carbonate fractured reservoirs, polymers need to be carefully selected for compatibility with the formation conditions. To select the appropriate polymers for the Tahe Oilfield, ZP-1 and ZP-2 were blended in a 1:1 ratio to study the effect of polymer blending ratios on the viscosity of the system. As shown in Figure 3a, the viscosity of the ZP-1 and ZP-2 blends gradually decreased as the temperature increased from 80 °C to 180 °C, a trend consistent with single-polymer behavior. This can be explained by the mechanism of polymer viscosity change with temperature, where an increase in temperature affects the speed of the polymer molecules’ random motion, thus influencing the molecular interactions and the viscosity of the polymer solution. At the same temperature, an increase in the blend ratio of the polymers resulted in a slight increase in viscosity. As shown in Figure 3b, at the same temperature, the viscosity of the blended system was slightly higher than that of the single polymer system. This is likely because the interaction between the polymers in the blend was increased, leading to tighter entanglement of the polymer molecules compared to the single polymer, which in turn increased the viscosity of the blended system.

#### 2.1.5. Optimization of Polymer Concentration and Type for Gel Composition

**Figure 4:**
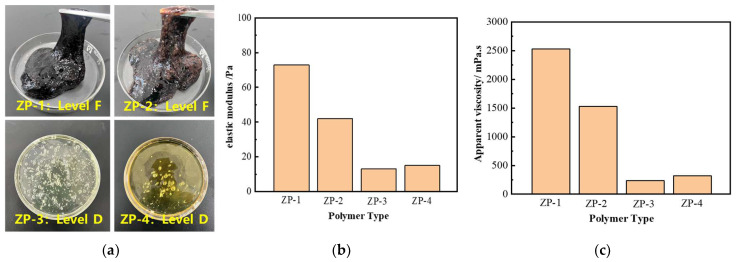
Crosslinking gels of different polymers: (**a**) morphology of crosslinking gels of different types of polymers; (**b**) storage modulus of crosslinking gels of different polymers; (**c**) apparent viscosity of crosslinking gels of different polymers.

We investigated the gelling of different types of polymers with phenolic aldehyde crosslinkers at 160 °C and observed the gelling effect as well as the gel storage modulus and apparent viscosity (Figure 4). The results show that the gelling effect of ZP-1 and ZP-2 polymers is the best, and the gel strength (storage modulus and apparent viscosity) is also higher. Therefore, ZP-1 and ZP-2 were selected as the preferred high-temperature crosslinking polymers.

We then used a blend of ZP-1 and ZP-2 at a total concentration of 1.0% and studied the gelling effect of the blended polymer with the same crosslinking agent under 180 °C conditions (Table 2). When the weight ratio of the two polymers was 1:1, the gel strength was the highest, reaching the H-level, making the ZP-1/ZP-2 blend (1:1 ratio) the optimal choice. The anti-high-temperature functional groups on the polymer chains of the two polymers intertwine, improving the compactness and high-temperature resistance of the gel structure.

**Figure 5:**
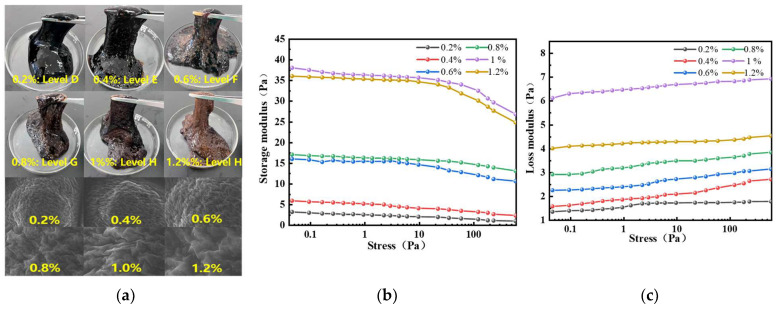
The effect of different polymer concentrations on the gelling system: (**a**) morphology of crosslinking gel at different polymer concentrations; (**b**) effect of different polymer concentrations on the storage modulus of the gelling system; (**c**) effect of different polymer concentrations on the loss modulus of the gelling system.

By fixing the crosslinking agent and urea-formaldehyde resin concentrations, the effect of different polymer concentrations on the gel gelling strength was investigated at 150 °C (Figure 5a). When the polymer concentration reached 1.0%, the gelling system’s storage modulus G′ and loss modulus G″ were maximized (Figure 5b,c), reaching 38 Pa and 6.5 Pa, respectively. The gelling strength of the system was optimal, and the gelling time was 6 h. Increasing the concentration further had little effect on improving the gelling strength, so the optimal polymer concentration was selected to be 1.0%.

**Figure 6:**
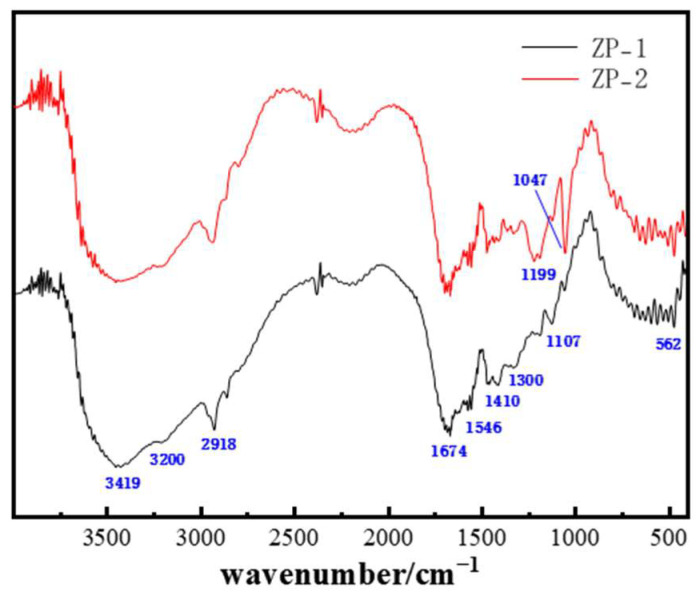
Infrared spectra of ZP-1 and ZP-2.

**Figure 7:**
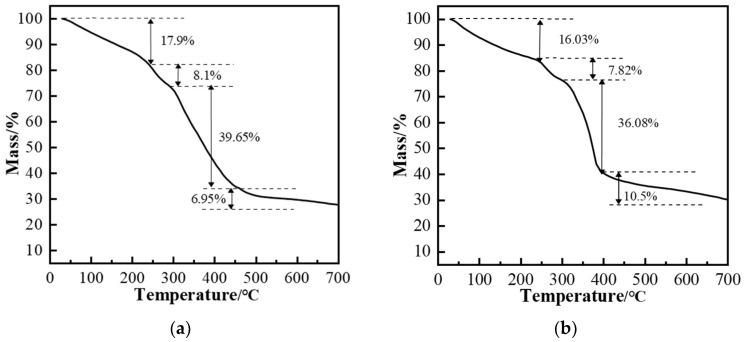
Thermogravimetric analysis curves of the polymers: (**a**) ZP-1; (**b**) ZP-2.

Figure 6 compares the infrared spectra of polymers ZP-1 and ZP-2. A strong and broad absorption peak around 3419 cm^−1^ indicates the presence of −NH_2_ or −COHN_2_ groups in the polymer structure. The absorption peak near 3200 cm^−1^ characterizes the presence of −COO− or −SO_3_− groups in the polymer structure. The absorption peak at 2918 cm^−1^ corresponds to the aliphatic C−H stretch in the polymer molecular structure. Strong absorption peaks at 1674 cm^−1^ and 1546 cm^−1^ are attributed to the C=O stretching vibration of the amide group. The strong absorption peak near 1410 cm^−1^ corresponds to the −CH_2_−CH_3_ absorption in the AMPS structure. The absorption peak near 1300 cm^−1^ corresponds to the C−N stretching vibration in the NVP structure. The strong absorption peak around 1199 cm^−1^ is related to the C=O structure, and the absorption peak near 1047 cm^−1^ corresponds to the S=O group. The absorption peak near 562 cm^−1^ corresponds to the −SO3H group. Based on the infrared analysis, it can be concluded that polymer ZP-2 contains the AMPS structure, while polymer ZP-1 contains both AMPS and NVP structures. To assess the thermal stability of the polymers, thermogravimetric analysis (TGA) was conducted. Figure 7a,b show the TGA curves of polymers ZP-1 and ZP-2. The molecular weight of ZP-2 is slightly higher than that of ZP-1. Typically, higher-molecular-weight polymers have longer molecular chains and greater entanglement and interaction between molecular chains, which contributes to increased thermal stability. This explains the slightly higher residual mass of ZP-2 than ZP-1 at the high-temperature stage. The higher degree of hydrolysis means that the polymer chains contain more polar groups such as carboxyl groups (-COOH), which enhance intermolecular hydrogen bonding and also improve hydrophilicity. However, a higher number of polar groups may also result in thermal decomposition occurring more readily. Therefore, although ZP-2 has a slightly higher degree of hydrolysis, its thermal stability may not be significantly better than that of ZP-1. NVP was additionally introduced into ZP-1, which is a monomer with good thermal stability and solubility resistance properties. The introduction of NVP helps to improve the overall thermal stability of the polymer and allows for the formation of a higher amount of residual carbon in the TGA test. It can be observed that the inflection point of the curves is around 250 °C, indicating that both polymers begin to rapidly degrade their backbone structure (chemical bonds) when the environmental temperature reaches 250 °C. Therefore, it can be concluded that these polymers can maintain a relatively stable chemical structure under temperatures not exceeding 250 °C, demonstrating good high-temperature resistance.

### 2.2. Crosslinking Agent Type and Concentration Optimization

#### 2.2.1. Selection of Aldehyde Crosslinking Agents

A total polymer concentration of 1.0% (ZP-1 and ZP-2), with a phenolic crosslinking agent concentration of 0.3% and a urea-formaldehyde resin concentration of 15%, was used to investigate the effect of different aldehyde crosslinking agents on the gelling performance of the system at 150 °C.

**Figure 8:**
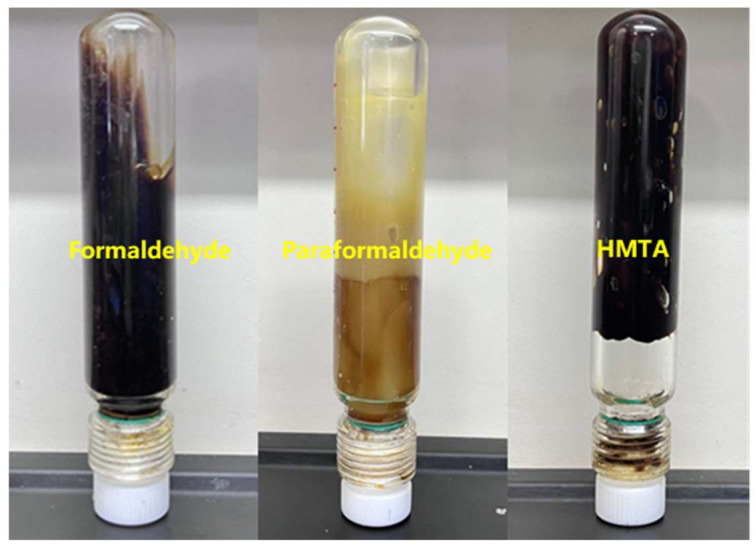
Comparison of gelling with three types of aldehyde crosslinking agents.

As shown in Table 3 and Figure 8, when formaldehyde was used as the aldehyde crosslinking agent, the gelling time of the system was very short, and the gel completely formed within about 1 h. This was mainly due to the rapid release of formaldehyde, which directly participated in the crosslinking reaction, leading to a very fast reaction rate. The gel strength quickly increased, but the rapid reaction rate made it difficult for the polymer to form a tight crosslinking structure with the crosslinking agent, resulting in a gel with obvious brittleness and rapid hydrolysis within 4 h. When paraformaldehyde was used, the gel system did not form a gel. Paraformaldehyde hydrolyzed to release formaldehyde at high temperatures, but the release rate of formaldehyde was slow. At prolonged high temperatures, the polymer chains underwent localized scission and hydrolysis, making it difficult to form stable crosslinking structures with the crosslinking agent before the polymer chains broke. The gelling process involved a pre-crosslinking stage, where microgel precursors were formed with the crosslinking agent. At 150 °C, the formaldehyde release rate from paraformaldehyde was insufficient to form polymer precursors before hydrolysis occurred, so no significant strength change was observed in the gel solution, and it completely hydrolyzed after 24 h of aging.

**Figure 9:**
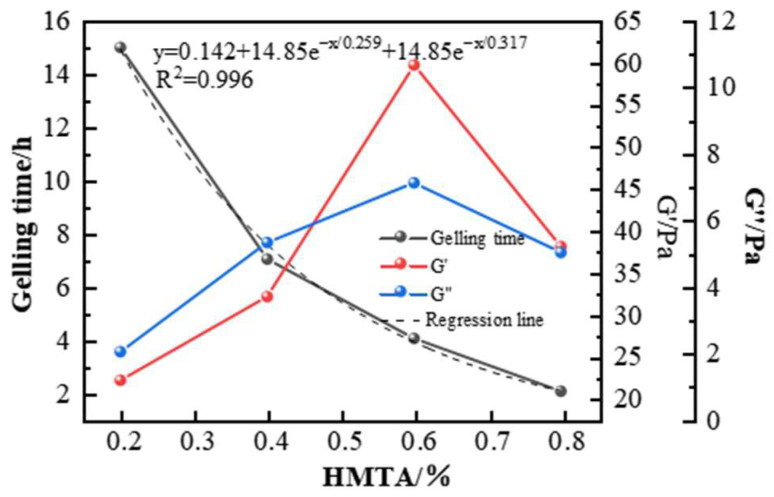
Effect of HMTA concentration on gelling time and storage modulus of the gel system.

**Figure 10:**
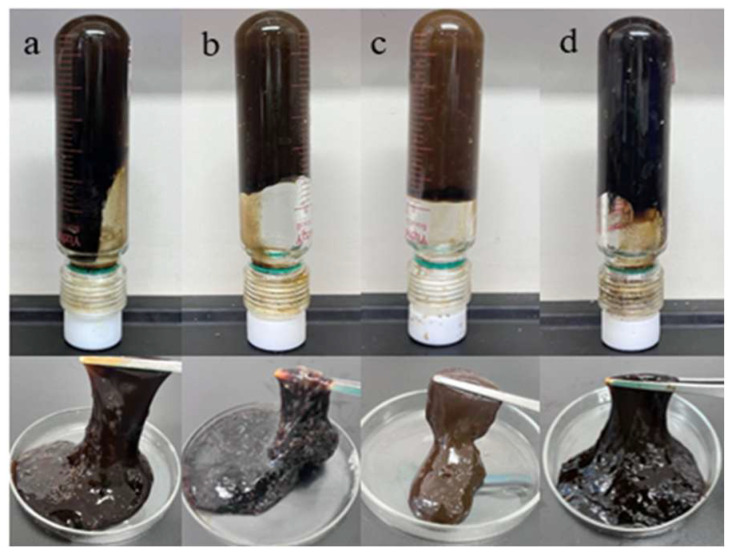
Gelling status of different HMTA concentrations: (**a**) 0.2%, (**b**) 0.4%, (**c**) 0.6%, (**d**) 0.8%.

When HMTA decomposed at high temperatures, it released formaldehyde at a moderate rate, providing a moderate crosslinking speed. This allowed the formation of a tight crosslinking structure before the polymer chains broke. The gel strength reached level H, and in bottle tests, it showed ideal wall-hanging performance. HMTA was therefore selected as the preferred aldehyde crosslinking agent. The effect of HMTA concentration on gelling performance was further investigated, and the experimental results are shown in Figure 9 and Figure 10. When the total polymer concentration remained constant at 1%, HMTA concentrations between 0.2 and 0.8% at 150 °C were able to form gel systems with different strengths. As the crosslinking agent concentration increased, the gelling time decreased, ranging from 2 to 15 h. At 0.2% HMTA, the gel strength was weak, only reaching E level. Within the 0.2–0.6% concentration range, as the HMTA concentration increased, the strength of the system significantly improved. At 0.6% HMTA, the gel strength reached level H, and the storage modulus and loss modulus of the gel system were maximized at 52 Pa and 9 Pa, showing excellent viscoelastic properties. However, a performance inflection point occurs after the concentration exceeds 0.6%, and the strength of the gel system decreases. This happens because when the concentration of the crosslinking agent is too high, the reaction rate is also greatly increased, and the gel system will experience excessive crosslinking phenomena, resulting in an increase in the proportion of network defects. Under this condition, it is difficult to form a stable crosslinking structure [34], which is manifested in the composite modulus value decreasing by 12~15%. Based on the principle of performance optimization, the preferred HMTA concentration is 0.6%.

#### 2.2.2. Phenolic Crosslinking Agent Selection

**Figure 11:**
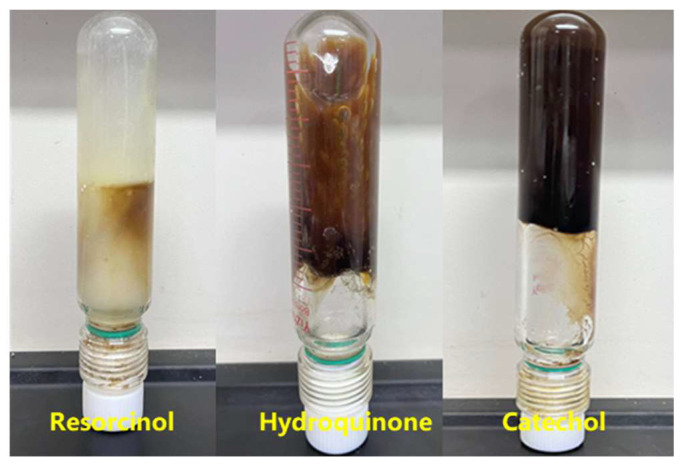
Comparison of gelling with three phenolic crosslinking agents.

The polymer concentration was set at 1.0%, HMTA crosslinking agent concentration at 0.6%, and resin curing agent concentration at 15%. The concentration of three phenolic crosslinking agents was 0.3%, and the experiments were conducted at 150 °C to study the effects of these crosslinking agents on the gelling performance of the system. The experimental results are shown in Table 4 and Figure 11.

**Figure 12:**
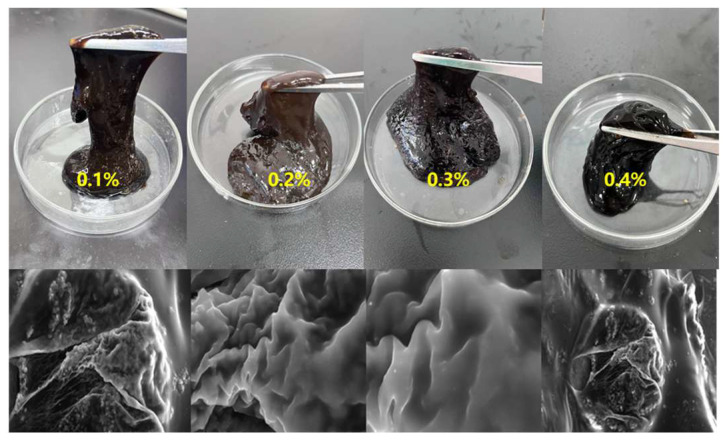
Gelling states at different phenolic crosslinking agent concentrations.

**Figure 13:**
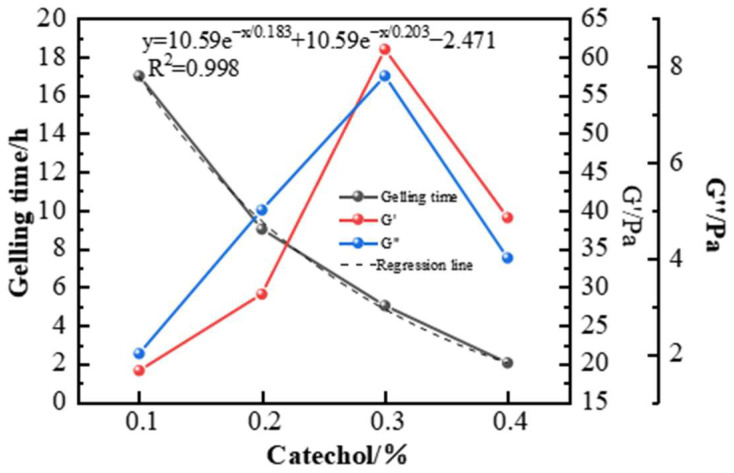
Effect of catechol concentration on gelling time and storage modulus of the gel system.

When catechol was used as the phenolic crosslinking agent to prepare the high-temperature polymer gel system, its gel strength was higher than that of the gel systems prepared with resorcinol and hydroquinone. The gel system exhibited the best wall-hanging performance, making catechol the preferred phenolic crosslinking agent. In the experiments at 150 °C, the effect of different catechol concentrations on the gelling performance is shown in Figure 12 and Figure 13. As the concentration of catechol increased, the gelling time decreased, and the gel strength first increased and then decreased. At a concentration of 0.3%, the gel system’s storage modulus and loss modulus reached their maximum values, 63 Pa and 9 Pa, respectively. The gel strength was substantial, and the gel system showed excellent wall-hanging performance in the bottle test. When the phenolic crosslinking agent concentration exceeded 0.3%, the elastic modulus and loss modulus of the gel system decreased, consistent with the phenomenon observed in aldehyde crosslinking agent experiments. Over-crosslinking in the system led to a decline in gel performance. Therefore, a catechol concentration of 0.3% is preferred.

### 2.3. Selection of Resin Curing Agent Type and Concentration

**Figure 14:**
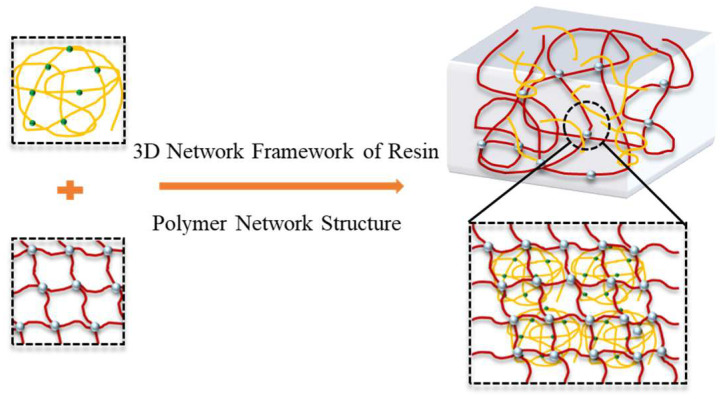
Resin network skeleton combined with polymer network structure schematic.

To enhance the stability of gel blockers in high-temperature environments, a resin curing agent (urea-formaldehyde resin) is added to the gel blocker. Under certain temperatures, it undergoes a self-polymerization curing reaction. The reactive functional groups in the resin, such as −CH_2_OH, −NH−, and −NH_2_, further react, crosslinking the resin to form a three-dimensional network structure, resulting in a hybrid third-body structure, i.e., a resin network skeleton (Figure 14). This structure significantly enhances the gel’s temperature resistance and gelling strength, thereby improving the gel’s high-pressure plugging capacity for large fracture loss after curing. A polymer concentration of 1.0%, crosslinking agent hexamethylenetetramine (HMTA) concentration of 0.6%, and 0.3% of phenolic crosslinker are used, with a gelling temperature of 150 °C. The effect of urea-formaldehyde resin dosage on the gel strength is examined.

**Figure 15:**
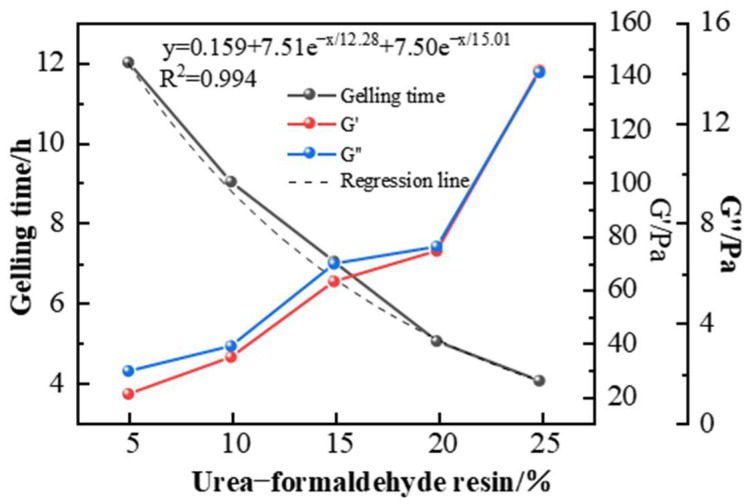
The effect of urea-formaldehyde resin concentration on gelling time and storage modulus in the gel system.

**Figure 16:**
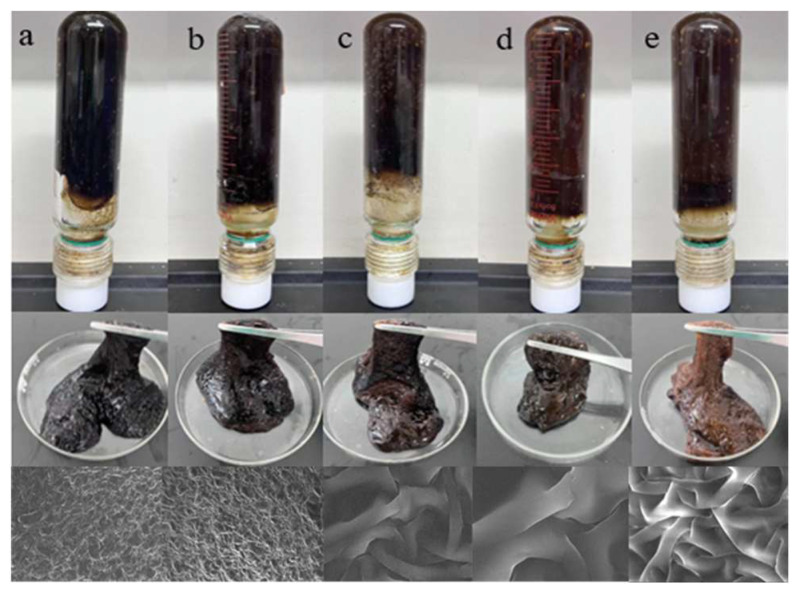
Gelling state of different resin curing agent concentrations: (**a**) 5%, (**b**) 10%, (**c**) 15%, (**d**) 20%, (**e**) 25%.

Figure 15 and Figure 16 show that when the polymer and crosslinking agent concentrations are kept constant, using urea-formaldehyde resin at concentrations ranging from 5% to 25% forms gel systems of varying strength with the polymer at 150 °C. As the resin concentration increases, the gelling time decreases, ranging from 4 to 12 h, and the gel strength continues to increase. At a resin concentration of 25%, the storage modulus and loss modulus reached 142 Pa and 14 Pa, respectively, satisfying the plugging requirements. However, excessively high resin concentrations damage the viscoelasticity of the gel and cause noticeable dehydration. Therefore, a urea-formaldehyde resin concentration of 25% is preferred.

### 2.4. Gel Plugging System Construction and Characterization

Based on the above research, the gel plugging system formulation is: 0.5% ZP-1 + 0.5% ZP-2 + 0.6% HMTA + 0.3% phenolic crosslinker + 25% resin curing agent.

**Figure 17:**
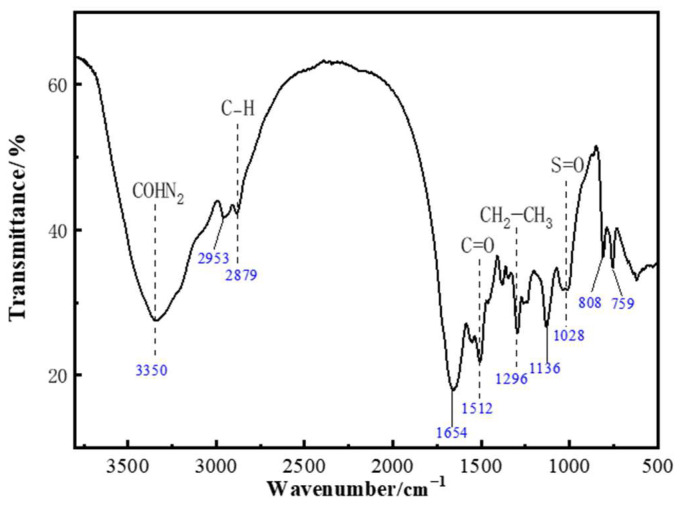
Infrared structural characterization of the polymer gel system.

As shown in Figure 17, the different structural units of the polymer gel system were characterized by infrared analysis. The strong and broad absorption peak around 3350 cm^−1^ indicates the presence of −NH_2_ or −COHN_2_ groups in the polymer structure. The absorption peak around 2953 cm^−1^ characterizes the presence of −COO− or −SO_3_− groups. The absorption peak at 2879 cm^−1^ corresponds to the aliphatic C−H absorption in the polymer molecular structure. Strong absorption peaks at 1654 cm^−1^ and 1512 cm^−1^ correspond to C=O stretching vibrations in the amide group. The strong absorption peak at 1296 cm^−1^ is attributed to the −CH_2_−CH_3_ absorption peak. The stretching vibration peak at 1136 cm^−1^ corresponds to the C−O structure, and the absorption peak at 1028 cm^−1^ corresponds to the S=O structure. Peaks at 808 cm^−1^ and 759 cm^−1^ correspond to the −SO_3_H absorption peak. The infrared spectrum indicates that the phenolic resin crosslinker forms a crosslinking structure with the polymer, synthesizing the target polymer gel system.

### 2.5. Analysis of Polymer Gel-Forming Mechanism

**Figure 18:**
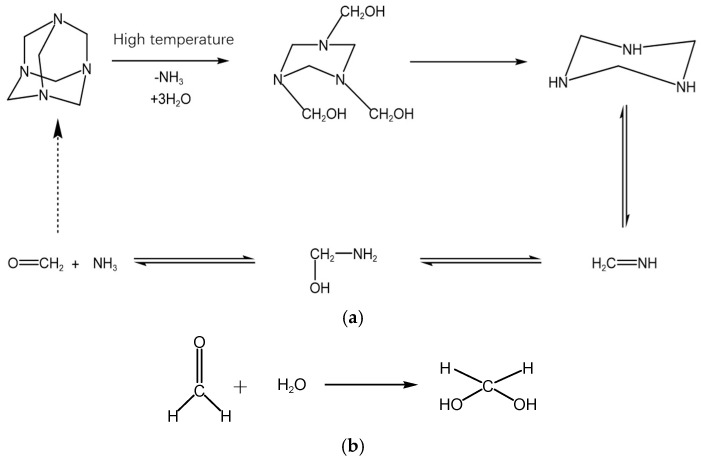

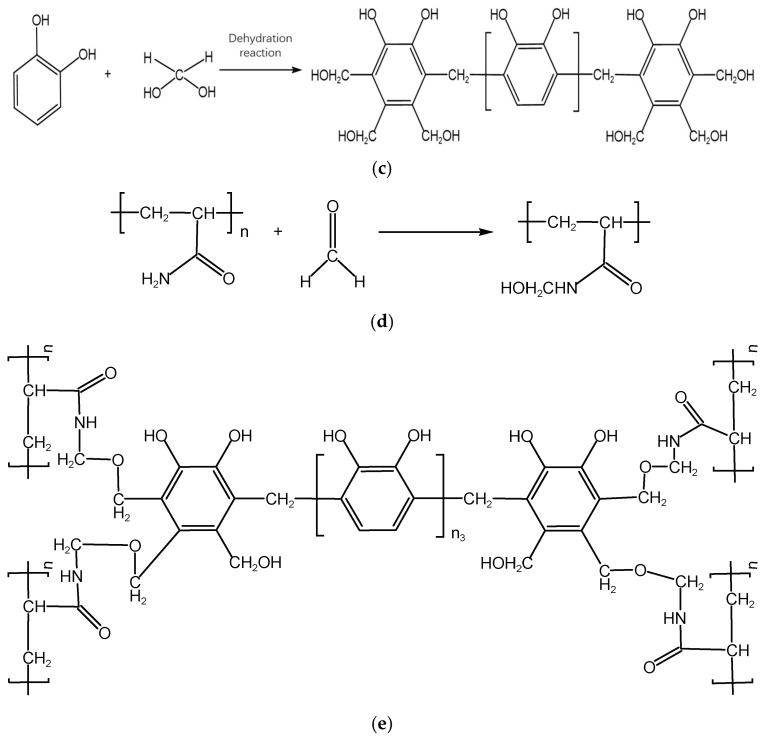
Crosslinking reaction mechanism analysis diagram: (**a**) Under the conditions of high temperature (>100 °C) and acidic environment (pH = 4~6), HMTA gradually pyrolyzes to generate formaldehyde and ammonia; (**b**) formaldehyde released is converted to the active intermediate methylene glycol by hydration; (**c**) etherification−condensation cascade reaction of methylene glycol molecules with catechol; (**d**) hydroxymethylation modification of the −CONH_2_ group with methyl glycol; (**e**) synthesis of gel system by dehydration condensation reaction between phenolic system and hydroxymethylated HPAM.

Based on the regulation laws of different phenolic crosslinking agent systems on polymer gelling properties, this subsection analyzes their gel-forming mechanism in depth. Take the hexamethylenetetramine (HMTA) crosslinking system as an example. Under the conditions of high temperature (>100 °C) and acidic environment (pH = 4~6), HMTA gradually pyrolyzes to generate formaldehyde and ammonia; the reaction formula is shown in Figure 18a. Then, the released formaldehyde is converted to active intermediate methylene glycol by hydration; the reaction formula is shown in Figure 18b. Then, with the methylene glycol molecule on the one hand, the etherification-condensation cascade occurs with catechol on the other. As the etherification-condensation cascade reaction occurs, catechol-1,2-dimethyl ether intermediate and part of the phenolic resin crosslinking agent are formed in turn; the reaction formula is shown in Figure 18c. At the same time, part of the −CONH_2_ group on the polyacrylamide (HPAM) will be hydroxymethylated as the higher activity of the methyl glycol modification occurs; the reaction formula is shown in Figure 18d. Finally, the phenol aldehyde system and hydroxymethylated HPAM are synthesized into a gel system by a dehydration condensation reaction; the reaction formula is shown in Figure 18e.

## 3. Physicochemical Properties of the Chemical Gel Plugging System

### 3.1. Shear Resistance of Gel Plugging System Solution

A HAAKE RS6000 rheometer is used, with shear rates ranging from 0.1 to 100 s^−1^ and a temperature range of 80 °C to 150 °C, to investigate the effect of shear rate on the viscosity of the polymer gel solution using the HAAKE Mars60 rheometer’s shear module. The change in the gel solution viscosity with shear rate is plotted.

**Figure 19:**
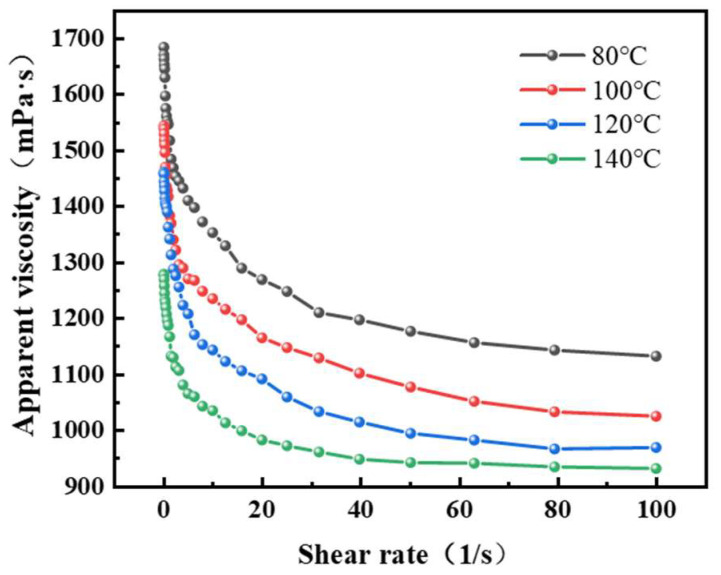
Relationship between the apparent viscosity of the gel solution and shear rate at different temperatures.

From the data in Figure 19, it can be observed that when the shear rate is below 20 s^−1^, the apparent viscosity of the gel solution gradually decreases with an increase in shear rate. Once the shear rate exceeds 20 s^−1^, the apparent viscosity of the gel solution remains relatively stable. This is mainly because under static conditions when the gel solution is not influenced by external forces, the polymer macromolecular chains can fully relax, and entanglements between the molecular chains occur, resulting in higher viscosity. However, when the gel solution is subjected to external shear forces, the polymer molecules coil, and the entanglements between molecules are easily disrupted, causing a decrease in viscosity. After a certain shear rate, the structure formed by the macromolecules, which are fully extended into irregular molecular coils in the aqueous solution, is destroyed. At this point, further increases in shear rate have little effect on viscosity. The test data show that at temperatures of 80 °C and 150 °C, the apparent viscosities at a shear rate of 0.1 s^−1^ are 1684 mPa·s and 1460 mPa·s, respectively. At a shear rate of 100 s^−1^, the apparent viscosities are 1132 mPa·s and 931 mPa·s, respectively. The apparent viscosity of the gel solution shows a minimal decrease with shear rate, indicating good shear resistance. Furthermore, under the same shear rate conditions, as the temperature increases, the apparent viscosity of the gel solution gradually decreases. However, at 150 °C and a shear rate of 100 s^−1^, the apparent viscosity of the gel solution remains above 950 mPa·s, which is 80.57% of the viscosity at 80 °C, indicating good high-temperature resistance of the gel solution.

### 3.2. Thixotropic Properties of the Gel Plugging System Solution

Thixotropy refers to the phenomenon where the apparent viscosity of a fluid exhibits a lag due to differences in the rates of internal structure destruction and recovery under the same shear force. As the gelling time increases, the polymer chains and crosslinkers in the gel system continue to undergo crosslinking reactions, and the extent of internal structural changes can be characterized by thixotropic experiments.

**Figure 20:**
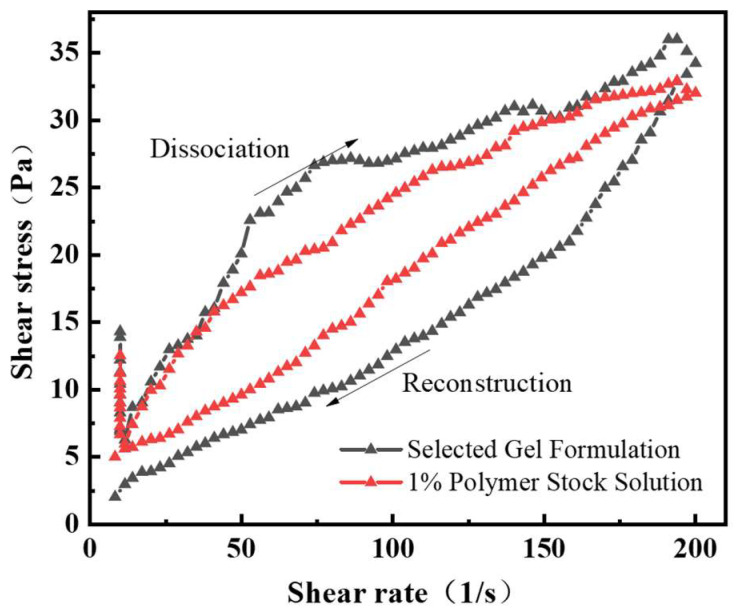
Shear stress versus shear rate relationship of the gel solution.

The device for thixotropic measurements is the same as the one used for rheological measurements. The sample is sheared at a certain temperature, with the shear rate increasing from 0 to a constant value, then gradually decreasing to 0. The stress as a function of shear rate is measured, and the closed shear stress versus shear rate curve is obtained, which is the thixotropic loop and can be used to characterize thixotropic properties. Thixotropic solutions are generally very sensitive to the initial conditions. To minimize this effect, the sample is pre-sheared before measuring the thixotropic loop area. A pre-shear rate of 10 s^−1^ is applied to the sample solution before the measurement, which then involves a cycle of increasing (10–200 s^−1^) and decreasing (200–0 s^−1^) shear rates. As shown in Figure 20, the apparent viscosity of the gel solution decreases initially with an increase in shear rate and then stabilizes. Under the shear rate cycle, there is a lag between the breakdown and reconstruction of the solution, and the selected thixotropic gel system shows a larger lag area than the polymer mother liquid, indicating that the gel solution exhibits excellent thixotropy.

### 3.3. High-Temperature Gelling Performance of the Gel Plugging System

**Figure 21:**
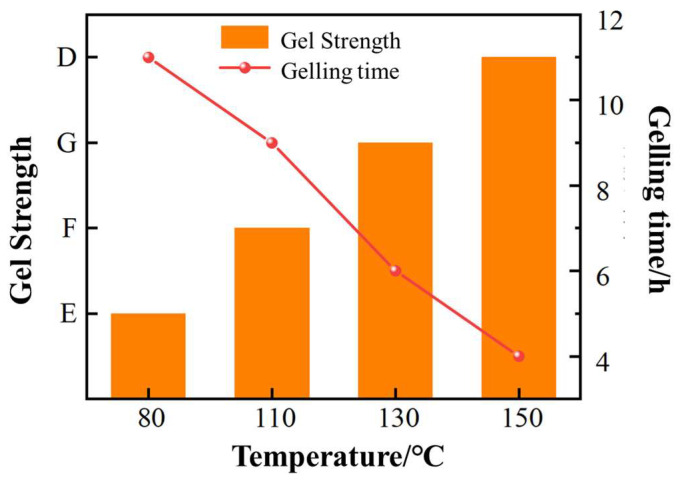
The effect of different temperatures on the gelling time of the gel system.

Temperature is not only a key factor affecting the gelling time and gel strength of the gel system, but also an important factor influencing its high-temperature stability. To examine the effect of different reaction temperatures on gelling time, the experimentally optimized gel system formulation is used as the high-temperature underground crosslinking polymer gel system. The temperature range studied is from 80 °C to 150 °C, and the experimental results are shown in Figure 21. As the gelling temperature increases from 80 °C to 150 °C, the gelling time is shortened from 8 h to 4 h.

As the reaction temperature increases, the effective collision opportunities between the polymer molecules and the organic phenolic crosslinking agents increase, which accelerates the crosslinking speed, thus shortening the gelling time. On the one hand, at high temperatures, the polymer undergoes intense hydrolysis and even high-temperature degradation at higher temperatures. On the other hand, under ultra-high temperatures, the crosslinking speed of the phenolic resin system increases, but complex crosslinking reactions still occur between the polymer and the crosslinking agent. The relationship between the gelling time and reaction temperature of the polymer gel system roughly satisfies the Arrhenius equation:

GT = Ae^Ea/RT^ (1)

where:

GT = gelling time (in hours);

A = frequency factor for effective collisions between active molecules (in hours);

E_a_ = apparent activation energy between active molecules and the reacting molecules (in kJ/mol);

R = universal gas constant (in kJ/(mol·K));

T = reaction temperature (in Kelvin).

Rearranging Equation (1), we get the following linear relationship between ln(GT) and 1/T:

lnGT = E_a_/RT + lnA (2)

**Figure 22:**
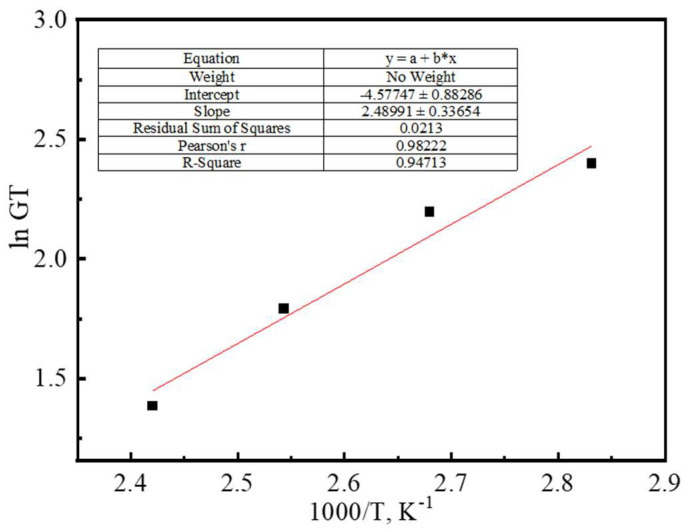
Arrhenius curve of the crosslinking reaction of the polymer gel system under different reaction temperatures.

The relationship curve obtained from the experimental data fitting is shown in Figure 22, and the slope of the line is 2489.9. From Equation (2), we obtain:

E_a_/R = 2489.9 (3)

Thus, the apparent activation energy E_a_ of this polymer gel system is 20.7 kJ/mol, the apparent activation energy in the typical range of physical crosslinking systems (10–50 kJ/mol), indicating that the reaction is mainly controlled by weak interactions such as hydrogen bonding. The low apparent activation energy (E_a_ = 20.7 kJ/mol) suggests that the gel network is mainly dependent on physical crosslinking, and its reversibility leads to a temperature-sensitive property, confirming that temperature regulation of crosslinking density follows the principle of constraints on thermodynamic parameters.

**Figure 23:**
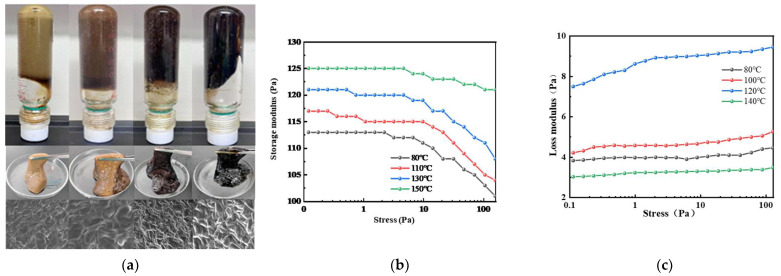
The effect of different temperatures on the storage and loss moduli of the gel system: (**a**) gelling state of the gel system at different temperatures: 80 °C, 110 °C, 130 °C, 150 °C; (**b**) the effect of different temperatures on the storage modulus of the gel system; (**c**) the effect of different temperatures on the loss modulus of the gel system.

From Figure 23, it can be seen that for the gel system under the same stress conditions at different temperatures, the elastic modulus G″ is always much larger than the viscous modulus G″, indicating that the gel has good elasticity, which is conducive to effective plugging of the losing formation. At a gelling temperature of 150 °C, the gel system exhibits the maximum values for both G′ and G″. As the shear frequency increases, the maximum values of G′ and G″ reach 125 Pa and 3 Pa, respectively, forming a tight three-dimensional network structure with excellent viscoelastic properties. When the temperature rises to 150 °C, the G′ and G″ values of the gel system drop significantly because, under high-temperature conditions, the molecular thermal motion increases, and the collision reaction rate between the polymer molecules and phenolic crosslinking agents accelerates. This prevents full crosslinking and decreases the tightness of the crosslinking structure, leading to a reduction in gel strength.

### 3.4. High-Temperature Stability of Gel Plugging Systems

**Figure 24:**
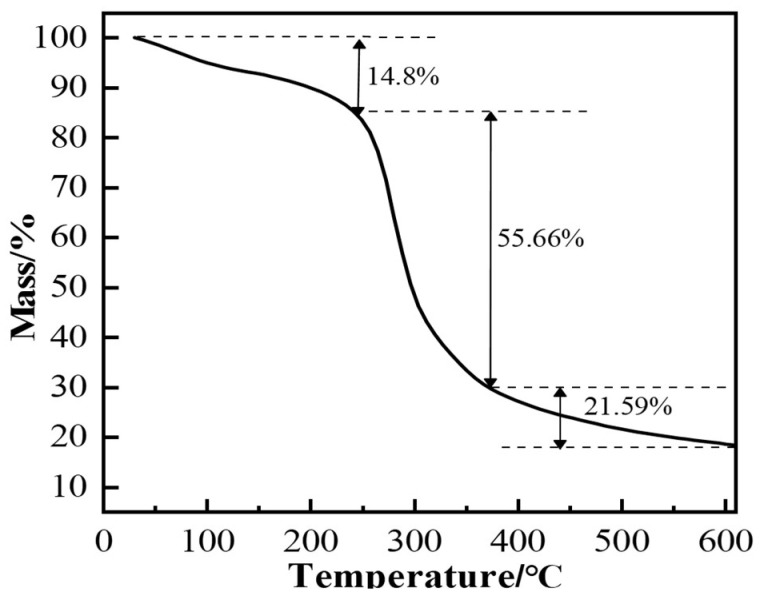
Thermogravimetric analysis (TGA) of the polymer gel material.

To evaluate the thermal stability of the polymer gel system, thermogravimetric analysis (TGA) was used to test the gel system’s thermal stability. Figure 24 shows the TGA curve of the gel sample. The curve shows an inflection point around 240 °C, indicating that the polymer gel system’s skeletal structure (chemical bonds) begins to break down rapidly when the temperature exceeds 240 °C. This suggests that the polymer gel system remains relatively stable in terms of chemical structure when the temperature does not exceed 240 °C.

**Figure 25:**
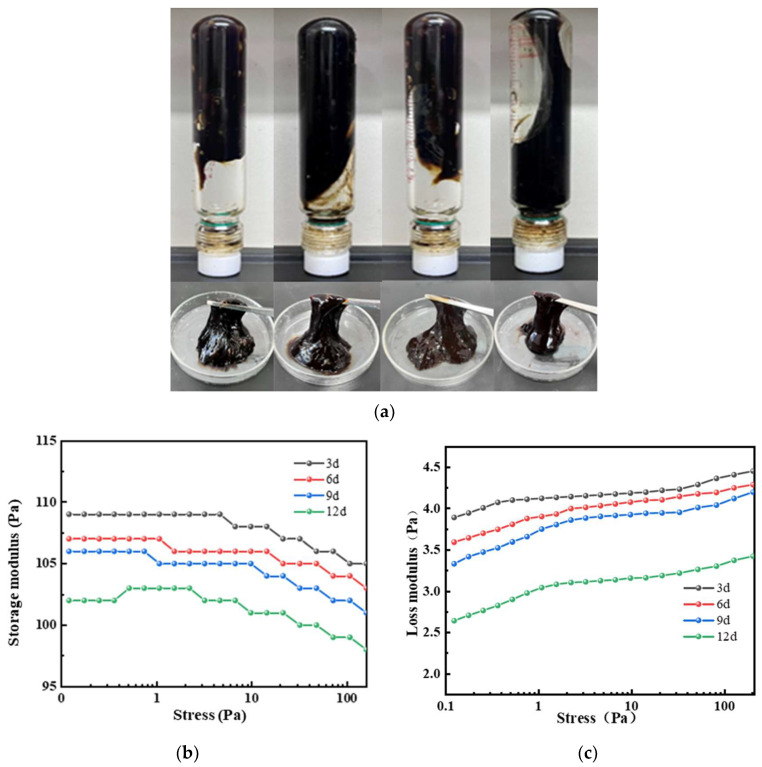

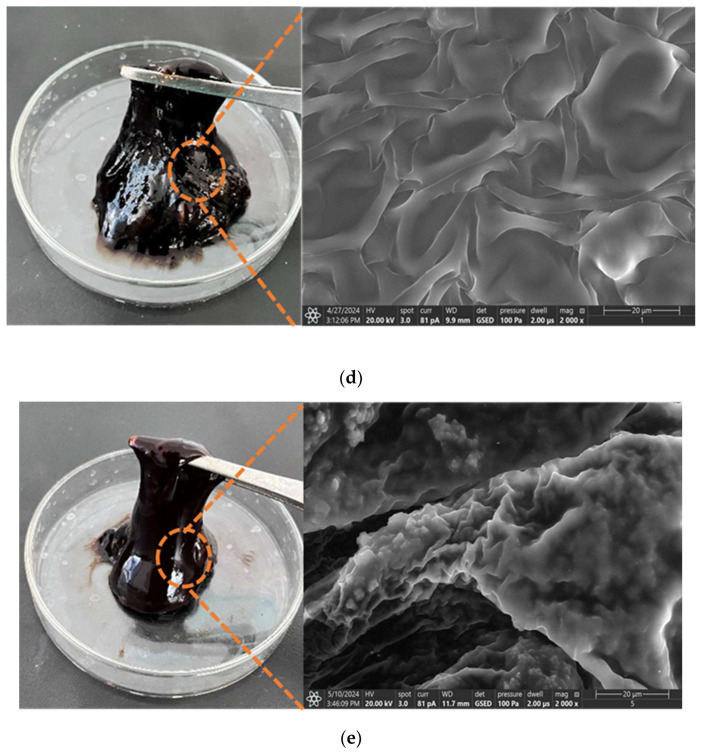
Aging of the gel system at 150 °C for different times: (**a**) gel states at different aging times: 3 days, 6 days, 9 days, 12 days; (**b**) effect of aging time on the storage modulus of the gel system at 150 °C; (**c**) effect of aging time on the loss modulus of the gel system at 150 °C; (**d**) microstructure of the gel after 3 days of aging at 150 °C; (**e**) microstructure of the gel after 9 days of aging at 150 °C.

Under high-temperature conditions, whether the gel system can maintain sufficient viscoelastic properties and strength over time directly determines the success of plugging. The temperature was set to 150 °C, and the sample was aged for 3, 6, 9, and 12 days. The gel’s storage modulus, loss modulus, and viscosity were measured with a rheometer to characterize the gel’s high-temperature stability. As seen in Figure 24 and Figure 25, when the aging time increased from 3 days to 12 days at 150 °C, both the viscosity and strength of the gel system showed a downward trend. After aging for 3 days at 150 °C, the G′ and G″ of the gel system were 109 Pa and 4.3 Pa, respectively. After 9 days of aging, the G′ and G″ were 105 Pa and 3.9 Pa, respectively. The gel system exhibited a slight reduction in G′ and G″ between 3 and 9 days, demonstrating excellent high-temperature stability. However, when the aging time exceeded 3 days, the viscosity and strength of the gel system significantly decreased. The gel system still formed a strong gel, maintaining a higher viscosity even under high-temperature aging conditions. After aging for 9 days, the viscosity retention rate was above 82%, showing excellent high-temperature stability. From the microstructure images of the gel at 150 °C after 3 days and 9 days of aging (Figure 25d,e), it can be observed that after aging for 3 days, the gel’s internal crosslinking structure was tight, with high gel strength and excellent viscoelastic properties. After aging for 9 days, the gel system showed some degradation, but it still maintained a tight crosslinking structure overall.

### 3.5. Salt Resistance of Gel Plugging Systems

**Figure 26:**
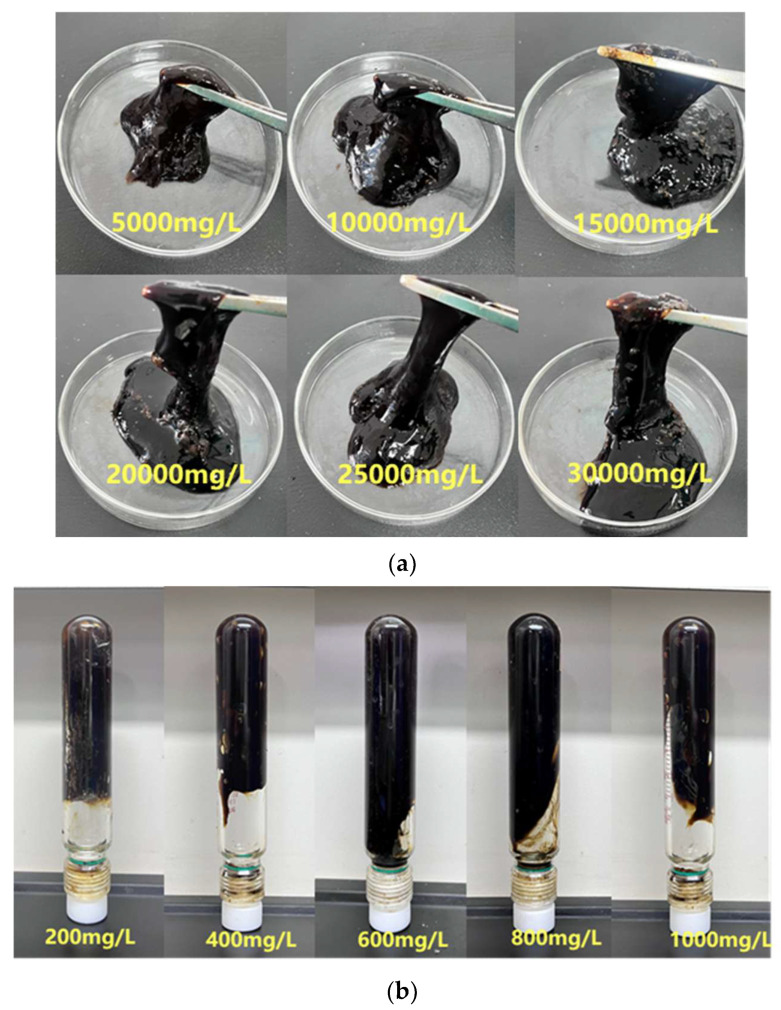

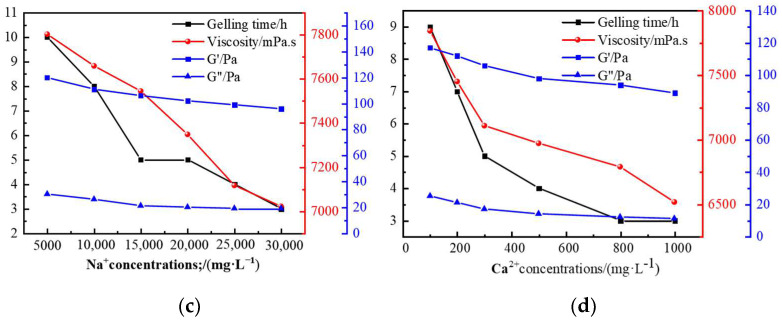
Gelling at different salt ion concentrations: (**a**) gelling states at different Na^+^ concentrations; (**b**) gelling states at different Ca^2+^ concentrations; (**c**) effect of Na^+^ concentration on the gelling performance of the gel system; (**d**) effect of Ca^2+^ concentration on the gelling performance of the gel system.

To study the impact of different concentrations of inorganic cations Na^+^ and Ca^2+^ on the gelling performance of the gel system, five sets of samples were prepared with Na⁺ concentrations of 5000, 10,000, 15,000, 20,000, 25,000, and 30,000 mg·L^−1^, and the gelling temperature was set to 150 °C. The gelling performance of the gel system was tested under different Na^+^ concentrations. As shown in Figure 26a,c, as the Na⁺ concentration increased from 5000 mg·L^−1^ to 30,000 mg·L^−1^, the gelling time decreased from 10 h to 3 h, and the gel system’s viscosity decreased to 7743 mPa·s. Compared to deionized water, monovalent cations compress the polymer’s double electric layer to a certain extent, reducing the repulsive force between polymer molecules and accelerating the crosslinking speed. Additionally, the polymer molecular clusters are compressed, reducing the reaction activity between the polymer and crosslinking agent molecules, resulting in a decrease in both viscosity and gel strength. As the Na^+^ concentration increased, the changes in the storage modulus and loss modulus of the gel system were relatively minor, indicating that Na^+^ had a limited effect on the gel system’s gelling strength. Under the high Na^+^ concentration of 30,000 mg·L^−1^, the storage modulus and loss modulus of the gel system were 96 Pa and 19 Pa, respectively, which was only slightly lower than the gel system’s storage and loss moduli in deionized water. The viscosity retention of the gel system was 91%, showing excellent resistance to Na^+^.

Similarly, five sets of samples were prepared with Ca^2+^ concentrations of 200, 400, 600, 800, and 1000 mg·L^−1^, and the gelling temperature was set to 150 °C to test the gel system’s gelling performance under different Ca^2+^ concentrations. As shown in Figure 26b,d, as the Ca^2+^ concentration increased from 200 mg·L^−1^ to 1000 mg·L^−1^, the gelling time decreased from 9 h to 3 h, and the gel system’s viscosity decreased to 6515 mPa·s. Compared to deionized water, the increase in Ca^2+^ concentration caused a greater compression of the polymer’s double electric layer. This decreased the solubility of the temperature-resistant polymer, reduced the viscosity of the polymer solution, and lowered the reaction activity between the polymer and crosslinking agent molecules, resulting in a decrease in both viscosity and gel strength. The viscosity retention rate reached 82%.

### 3.6. Gel Degradation Performance of Gel Plugging Systems

**Figure 27:**
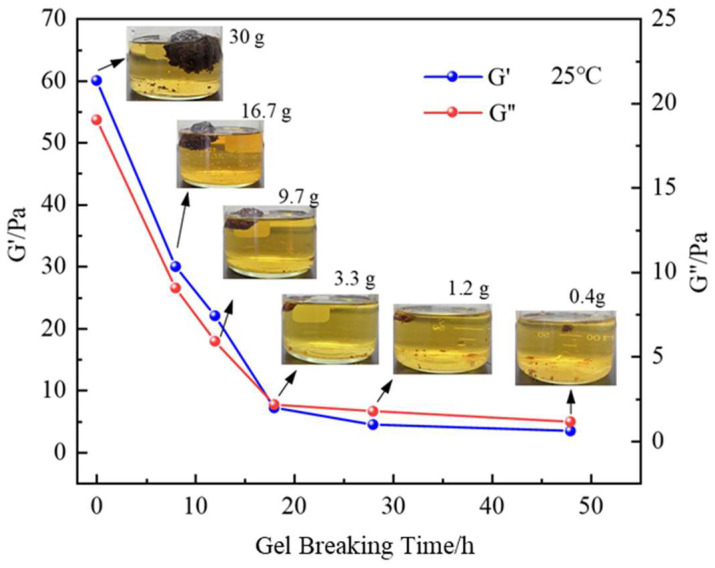
Changes in the gel over time after soaking in a 15% ammonium persulfate solution.

High-temperature and high-pressure conditions pose significant challenges for rapid gel degradation in wellbore and fracture. The gel system studied in this paper is easily degraded in a short time, solving the problem of controlling the gel degradation time and complexity of degradation under high-temperature conditions in traditional gel systems. The chosen gel degradation agent is widely available and inexpensive. As shown in Figure 27, at room temperature, after soaking in a 15% ammonium persulfate solution for 12 h, the gel volume significantly decreased, and its mass decreased from 30 g before degradation to 9.7 g. After 24 h, the gel system’s degradation was almost complete, with the mass reduced to 10% of its original value, and the gel system’s strength decreased significantly. The storage modulus and loss modulus were 12 Pa and 2 Pa, respectively. Under the 150 °C condition, after adding the same concentration of degradation agent, the gel degradation speed was further accelerated. After soaking in a 15% ammonium persulfate solution for 12 h, the mass decreased from 30 g to 2.1 g, and the solution color changed to dark yellow, with significant degradation of the gel system. The storage modulus and loss modulus were 11 Pa and 2 Pa. After soaking in a 15% ammonium persulfate solution (100 mL solution and 30 g gel block) for 12 h at 150 °C, the gel degradation rate exceeded 70%, and after 24 h, the degradation rate reached 99.8%. The strong oxidant interacts with the polymer chains that form the gel’s framework, causing the molecular chains to break, allowing for rapid degradation in a short time. Therefore, after using this gel for wellbore plugging, ammonium persulfate aqueous solution can be used to degrade the gel.

**Figure 28:**
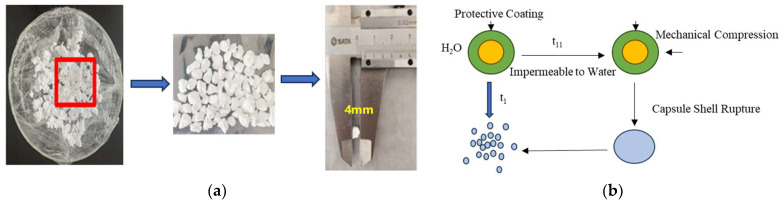
Capsule degradation agent: (**a**) physical image of the capsule degradation agent; (**b**) schematic of the release process of the capsule degradation agent.

Capsule-type degradation agents (Figure 28a) can be directly added to the gel plugging system solution. Once the gel forms, the capsules slowly release the degradation agent at a certain temperature to degrade the gel (Figure 28b). The “core-shell” structure microcapsules have a core material of aminomethanesulfonic acid and a shell of thermoplastic resin. The release time and rate of the microcapsule degradation agent can be controlled by adjusting the capsule’s shell thickness, shell material, medium temperature, and core material.

**Figure 29:**
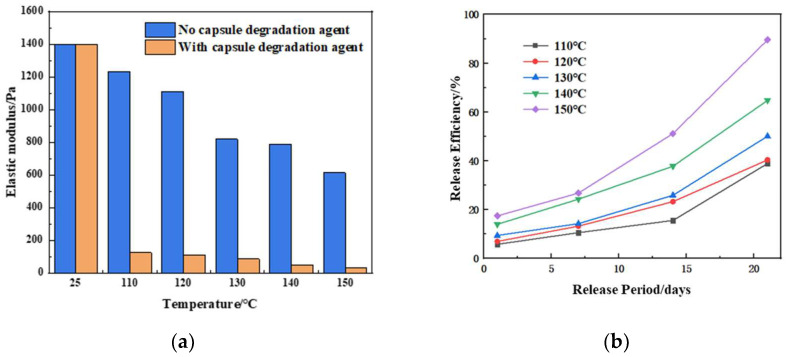
Capsule degradation agent at different temperatures: (**a**) elastic modulus of the gel after degradation with the capsule degradation agent at different temperatures; (**b**) release rate curve of the capsule degradation agent at different temperatures.

Using a 10% capsule degradation agent for degradation, the elastic modulus (G′) of the gel after degradation at different temperatures was measured. As shown in Figure 28a, after 15 days of using the capsule degradation agent at 150 °C, the elastic modulus of the gel was about 630 Pa when degradation began. After degradation, the elastic modulus rapidly decreased to approximately 33 Pa, weakening the gel strength. At 150 °C, the degradation agent could last for 15 days, with a release rate of 50.8%. After 21 days, the release rate was greater than 90% (Figure 29b). As the temperature increased, the capsule shell softened and its strength decreased, allowing the core degradation agent material to be released quickly [35].

## 4. Conclusions

(1) By optimizing key additives such as high-temperature-resistant crosslinking polymers and crosslinking agents, and determining the optimal types and ratios of the main agent and crosslinking agent, the formulation for a chemical gel plugging agent system resistant to temperatures up to 150 °C was developed: 0.5% ZP-1 + 0.5% ZP-2 + 0.6% HMTA + 0.3% phenol + 25% resin curing agent.
(2) The apparent viscosity of the chemical gel plugging agent solution shows a significant lag between the breakdown and reconstruction of the solution during the shear rate cycles, with a large lag area, indicating the excellent thixotropic behavior of the system. The system is formed into a gel at 150 °C, the storage modulus is 125 Pa, the apparent viscosity is about 7500 mPa·s, and the viscosity retention rate of the gel system is more than 82% after aging at 150 °C for 9 days, which shows excellent high-temperature stability performance.
(3) For an in-depth investigation of the influence mechanism of multivalent cations (Na^+^, Ca^2+^) on the gel network, the concentration gradient experiment of Na^+^ and Ca^2+^ was constructed. The composite modulus level of 96 Pa versus 19 Pa was still maintained under the condition of high Na+ concentration of 30,000 mg/L, and the viscosity retention was kept at the level of 91%. With the increase of Ca^2+^ concentration, the gel formation time of the system was shortened from 9 h to 3 h, and the apparent viscosity decreased to 6515 mPa-s, with a viscosity retention of 82%. This indicates that the preferred gel system has excellent salt resistance.
(4) For the gel system at room temperature and 150 °C high-temperature conditions, with the use of a concentration of 15% ammonium persulfate-soaked gel block (fixed solution 100 mL and gel block 30 g) for 12 h, the gel had a breakage rate of more than 70%, and a breakage rate of 99.8% after 24 h. High-temperature conditions result in faster breakage of the gel and breakthrough of the chemical plugging agent of the bottleneck.

## 5. Experimental Materials and Methods

### 5.1. Experimental Materials

The polymer materials used were ZP-1 and ZP-2 from Shandong Nor Chemical Co., Ltd., Dongying, China; the molecular weights of ZP-1 and ZP-2 are 700–900 and 800–1000 Daltons, respectively. The aldehyde-based crosslinking agents used were formaldehyde and paraformaldehyde from Shanghai Aladdin Reagent Co., Ltd., Shanghai, China, and hexamethylenetetramine (HMTA) from Shanghai Macklin Biochemical Technology Co., Ltd., Shanghai, China. The phenolic crosslinking agents used were resorcinol, hydroquinone, and catechol from Shanghai Macklin Biochemical Technology Co., Ltd., Shanghai, China. Formaldehyde, paraformaldehyde, HMTA, resorcinol, hydroquinone, and catechol are pure AR. The resin curing agent used was urea-formaldehyde resin from Zibo Ocean Industrial Co., Ltd., Zibo, China. Deionized water prepared in the laboratory was used as the solvent.

### 5.2. Experimental Methods

#### 5.2.1. Preparation of Polymer Solution

Deionized water was used to prepare a 1.0% (*w*/*v*) solution of the polymer. The polymer solution was stirred thoroughly and aged at room temperature (25 °C) for 24 h. The stock solution was then diluted with deionized water to the required concentration for the experiment.

#### 5.2.2. Evaluation of Gelling Time and Gelling Effect of Polymer Gel System

The plugged ampoules were placed in a pre-set temperature constant temperature drying oven to allow the crosslinking reactions between different types of polymers and crosslinking agents to occur under constant temperature conditions. The gelling process was primarily observed using the Sydansk bottle test, where the gel state was monitored over time by observing the gel’s flow behavior. The time at which the gel strength reached D-grade was recorded as the initial gelling time. The time when the gel strength ceased to change under high-temperature conditions was recorded as the gelling time (GT).

**Figure 30:**
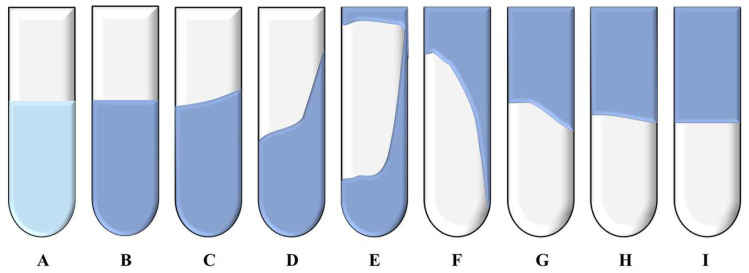
The code illustration of different gel strengths [10].

The gel system was classified into nine levels (A to I) based on different flow states, suspension states, and tongue-out states, as shown in Figure 30. The detailed descriptions of the gel strength ratings are as follows:

(A) Non-detectable gel: The viscosity of the gel system is almost the same as before preparation, and no obvious viscosity change can be seen with the naked eye.

(B) Gel with high flowability: The viscosity of the system slightly increases.

(C) Gel with moderate flowability: When the ampoule is inverted, most of the gel flows to the other end of the bottle.

(D) Gel with medium flowability: A small portion (<15%) of the gel cannot flow to the other end of the bottle when inverted.

(E) Almost non-flowing gel: A small amount of gel slowly flows to the other end of the bottle when inverted, but more than 85% of the gel cannot flow.

(F) High deformation but non-flowing gel: The gel system cannot flow to the bottom of the bottle when inverted.

(G) Medium deformation but non-flowing gel: The gel system can only flow to the middle of the bottle when inverted.

(H) Slight deformation and non-flowing gel: The gel cannot flow when the ampoule is inverted, and only a small part of the surface of the gel undergoes deformation.

(I) Rigid gel: The gel cannot flow when the ampoule is inverted, and the gel’s surface undergoes little or no deformation.

#### 5.2.3. Strength Testing of Polymer Gel System

The polymer gel system obtained under different gelling conditions was analyzed using a HAKKER Mars60 rheometer (Thermo Scientific, Waltham, MA, USA.) with a parallel plate testing system. The rotor model used was P35, and the gap between the plates was 0.052 mm. Dynamic rheological testing was conducted with shear mode and oscillation mode analysis, with a fixed frequency of 1 Hz. The shear rate ranged from 0.1 s^−1^ to 100 s^−1^, and the shear stress increased from 0.1 Pa to 100 Pa.

#### 5.2.4. High-Temperature Stability of Polymer Gel System

The long-term stability of the polymer gel system was semi-quantitatively evaluated by the gel dehydration rate. After evaluating the gelling effect, the sealed ampoules were placed in a constant temperature drying oven at 120–160 °C. Once the gel had formed, the ampoules were taken out every 3 days, and the weight of the gel after dehydration was measured using a balance. The dehydration rate (S) was calculated as the ratio of the dehydration amount of the gel to the initial mass of the gel.

#### 5.2.5. Microstructure Analysis of Polymer Gel System

The microstructure of the polymer gel system was analyzed using a JSM-7200 F Environmental Scanning Electron Microscope (ESEM). The gel sample was rapidly frozen in liquid nitrogen, and then the water in the sample was sublimated into gas using a vacuum freeze dryer. The sample was attached to the test stage with conductive adhesive tape, and gold powder was sprayed onto the surface of the sample to improve conductivity. The sample was then placed in the ESEM sample chamber, and various magnification images were obtained at room temperature using specialized software. Images with a white scale bar representing 10 μm at the center of the bottom of the sample were selected.

#### 5.2.6. Infrared Spectroscopy Characterization of Polymer Gel System

The molecular structure of the gel was characterized using a Fourier Transform Infrared Spectrometer (Shanghai Precision Instruments Co., Ltd., Shanghai, China.). The dried gel particles were ground into powder with potassium bromide (KBr) in a mortar. The mixed powder was then pressed into a pellet under high pressure, with the total mass of the gel powder accounting for 1.0% of the KBr powder mass. The scanning range was set from 400 to 4000 cm^−1^, and the number of scans was set to 35.

#### 5.2.7. Thermogravimetric Analysis of Polymer Gel System

The thermal stability of the gel’s molecular structure was measured using a Thermogravimetric Analyzer (TGA, TGA 2 SF) (METTLER TOLEDO, Columbus, OH, USA.). The instrument was preheated, and experimental parameters such as temperature and heating rate were adjusted. An empty crucible was placed first, and the mass was zeroed. Then, an appropriate amount of plugging agent was placed in the instrument. For all samples, the measurement pressure was 300 Pa, the flow rate was 20 cm^3^/min, the heating rate was 10 °C/min, and the temperature range was 30–600 °C.

#### 5.2.8. Pollution Resistance Evaluation of Polymer Gel System

When the gel solution is injected into the formation and comes into contact with high-salinity formation water, the gelling performance of the gel system is influenced by the mineralization degree. The gelling performance of the gel system was evaluated under different Na^+^ and Ca^2+^ concentrations. In practical field applications, the compatibility of the gel solution with the drilling fluid base slurry is an important factor to consider. The gel solution was mixed with drilling fluid at different volume ratios, and the gelling time and gelling strength of the gel system under drilling fluid mixing conditions were observed.

## Figures and Tables

**Table 1:** Basic properties of different polymers.

Polymer ID	Polymer Molecular Weight (Daltons)	Polymer Hydrolysis Degree (%)	Monomer Composition
ZP-1	700~900	10~15	AM/AMPS/NVP
ZP-2	800~1000	15~20	AM/AMPS
ZP-3	400~600	20–25	AM
ZP-4	500~700	20–25	AM

**Table 2:** Effect of polymer blending on gelling strength.

Polymer Type	Blend Ratio	Phenol Concentration (%)	Aldehyde Concentration (%)	Gelling Time (h)	Gelling Strength
ZP-1/ZP-2	1:2	0.2	0.4	14	E
	2:1	0.2	0.4	10	F
	1:1	0.2	0.4	8	H

**Table 3:** Effect of different aldehyde crosslinking agents on gelling time and thermal stability.

Aldehyde Crosslinker and Concentration (%)		Phenolic Concentration (%)	Gelling Time (h)	Gelling Effect and Thermal Stability
Formaldehyde	0.6	0.3	1	The gelling strength reaches G, followed by 5 h of high-temperature degradation.
Paraformaldehyde	0.6	0.3	Not gelled	/
HMTA	0.6	0.3	6	Gelling strength H, with dehydration less than 10% after 7 days.

**Table 4:** Effect of different phenolic crosslinking agents on gelling time and thermal stability of the gel system.

Phenolic Crosslinking Agent and Concentration (%)		Phenolic Concentration (%)	Gelling Time (h)	Gelling Effect and Thermal Stability
Resorcinol	0.3	0.6	8	Gel strength reached D; 12 h high-temperature degradation.
Hydroquinone	0.3	0.6	6	Gel strength G; dehydration < 10% after 7 days.
Catechol	0.3	0.6	6	Gel strength H; dehydration < 10% after 7 days.

## Data Availability

Data are contained within the article.
